# Effect of an (–)-Epicatechin Intake on Cardiometabolic Parameters—A Systematic Review of Randomized Controlled Trials

**DOI:** 10.3390/nu14214500

**Published:** 2022-10-26

**Authors:** Lisa Dicks, Zeina Haddad, Stefanie Deisling, Sabine Ellinger

**Affiliations:** Department of Nutrition and Food Sciences, Human Nutrition, University of Bonn, Meckenheimer Allee 166a, 53115 Bonn, Germany

**Keywords:** epicatechin intake, glucose and lipid metabolism, blood pressure, vascular function, inflammation, oxidative stress, cardiometabolic diseases, prevention, randomized controlled trials

## Abstract

Growing evidence exists that consumption of cocoa-rich food improves the parameters of cardiometabolic health. These effects are ascribed to cocoa flavanols, particularly to (–)-epicatechin (EC), a natural ingredient of cocoa. Hence, to evaluate if EC may explain the effects of cocoa, this systematic review aimed to provide an overview on randomized controlled trials (RCTs) investigating the impact of an EC intake on cardiometabolic biomarkers. For this, the Preferred Reporting Items for Systematic reviews and Meta-Analyses (PRISMA) 2020 statement was considered and the risk of bias (RoB) was assessed by using the Cochrane RoB 2 tool. In total, 11 studies were included examining parameters on vascular function, glucose/lipid metabolism, oxidative stress, inflammation, appetite sensations, and body weight before and after EC treatment. Except for a dose-dependent acute increase in flow-mediated dilatation (FMD) and in the peripheral arterial tonometry (PAT) index in healthy young adults, effects by EC treatment were not observed. For most trials, some concerns exist for overall RoB. Thus, EC intake may improve endothelial function in healthy young adults. For further parameters (mostly secondary outcomes), it remains unclear if EC has no effect or if this was not detectable. Unbiased RCTs on the impact of an EC intake are needed, which should also investigate the additive or synergistic effects of EC with other cocoa ingredients.

## 1. Introduction

Cardiovascular diseases (CVDs) are still the leading cause of death in modern societies worldwide. According to the World Health Organization (WHO), 17.9 million people died from CVDs in 2019, representing 32% of all deaths. Incidence as well as the progression of CVDs are strongly affected by behavioral risk factors such as physical inactivity and unfavorable diet, as these may lead to overweight/obesity, elevated levels of blood glucose and lipids, and increased blood pressure (BP) [1].

In this respect, cocoa is of particular interest, as various meta-analyses of randomized controlled trials (RCTs) have demonstrated that the regular intake of cocoa and cocoa products may increase flow-mediated dilatation (FMD) [2,3], insulin sensitivity [2,3,4], and high-density lipoprotein cholesterol (HDL-C) [2,3,4], while reducing BP [2,3,5], triglycerides (TGs) [2,4], low-density lipoprotein cholesterol (LDL-C) [2,3,6], *C*-reactive protein (CRP) [4], and selected biomarkers of oxidative stress [7]. Another meta-analysis of RCTs showed that cocoa consumption (≥30 g/d) for 4–8 wk may reduce body weight (BW) [8]. This is interesting with regard to the results of a recently published RCT that daily consumption of 100 g chocolate in the morning and/or evening reduces hunger, appetite for sweets, and *ad libitum* energy intake, while increasing physical activity and skin heat dissipation compared to no chocolate intake [9]. Cocoa products which provide ≥200 mg flavanols with a degree of polymerization (DP) of 1–10 per daily serving size were approved by the European Food Safety Authority (EFSA) health claim “cocoa flavanols (CF) help maintain the elasticity of blood vessels, which contributes to normal blood flow” [10]. An RCT with a double-blind placebo-controlled design including 21,442 adults aged ≥ 60 y has shown that the daily ingestion of a cocoa extract lowered the risk of CVD deaths by 27% and those of major cardiovascular events by 16% after a median intake for 3.6 y. This extract provided daily 500 mg CF (DP 1–7), among them 80 mg (−)-epicatechin (EC), but also other ingredients, e.g., methylxanthines (50 mg theobromine, 5 mg caffeine) [11].

The cardioprotective effects of cocoa are particularly ascribed to EC, the main monomeric flavanol in roasted cocoa beans and in cocoa products [12]. The bioavailability of EC is higher compared to other monomeric CF (e.g., (+)-epicatechin, (+)-catechin, (−)-catechin) [13]). Furthermore, the amount of EC ingested with cocoa products has shown to predict the decrease in BP in a metaregression analysis of RCTs [14].

Finally, biological plausibility exists that EC may contribute to cardiometabolic health. As reviewed recently [15,16,17,18], EC and its metabolites may decrease the production of reactive oxygen species (ROS), e.g., by mitigating the expression and the activity of NADPH oxidase and further ROS-generating enzymes. Moreover, EC can increase the availability of nitric oxide (NO) by stimulating endothelial nitric oxide synthase (eNOS) and/or by decreasing its degeneration through reaction with superoxide; this may contribute to vasodilation and a decrease in BP. Furthermore, EC can modulate redox-sensitive transcription factors (TFs) by activating nuclear factor E2-related factor 2 (Nrf2; a key regulator of antioxidant responses), while downregulating the proinflammatory nuclear factor kappaB (NF-κB). Oxidant-mediated activation of both c-Jun *N*-terminal kinase 1/2 (JNK) and inhibitor of nuclear factor kappaB (IκB) kinase (IKK) can inhibit the insulin-signaling cascade; this might be prevented by EC. Consequently, EC may improve glucose homeostasis by lowering inflammation and oxidative stress in the liver, muscle, and adipose tissue, and by increasing the secretion of insulin and incretins such as glucagon-like peptide-2 (GLP-2). Additionally, beneficial effects on serum lipids are possible, as EC can modulate the expression of transcription factors involved in triglyceride and cholesterol synthesis; EC may activate 5′-AMP-activated protein kinase, which stimulates the catabolism of lipids and glucose. In addition, EC may upregulate the expression of uncoupling protein-1 (UCP-1) that mediates the mitochondrial dissipation of energy by heat without the formation of adenosine triphosphate (ATP). This might contribute to a negative energy balance. However, knowledge on the potential mechanisms of action by EC is based on in vitro and animal studies; their clinical relevance has not been clarified yet.

Thus, the aim of this systematic review was to provide a systematic overview on RCTs that investigated whether an EC intake can modulate the parameters of cardiometabolic health (main outcomes: vascular function, glucose and lipid metabolism; additional outcomes: inflammation, oxidative stress, appetite sensations, BW). Moreover, the risk of bias (RoB) was assessed for each study to consider study limitations that could have biased the outcomes.

## 2. Materials and Methods

This systematic review was performed considering the Preferred Reporting Items for Systematic Reviews and Meta-Analyses (PRISMA) 2020 statement [19]. It was registered at the International Prospective Register of Systematic Reviews (PROSPERO) database on 24 December 2021 (registration ID: CRD42021293038).

### 2.1. Literature Search Strategy

A systematic literature search was conducted in the PubMed and Web of Science databases for RCTs, which investigated the effect of an EC intake on cardiometabolic health. For the literature search, the following Medical Subject Heading (MeSH) term was used: ((“epicatechin”) AND (“randomized controlled trial” OR “trial” OR “clinical trial”) AND (“cardiometabolic” OR “blood pressure” OR “prehypertension” OR “hypertension” OR “vasodilation” OR “endothelin-1” OR “nitric oxide” OR “arterial stiffening” OR “arterial stiffness” OR “pulse wave velocity” OR “vascular function” OR “endothelium-derived hyperpolarizing factor” OR “FMD” OR “antiatherogenic” OR “endothelial dysfunction” OR “atherosclerosis” OR “glucose” OR “insulin” OR “fructosamine” OR “HbA1c” OR “advanced glycation end products” OR “HOMA” OR “hypoglycemic” OR “hyperglycemic” OR “impaired fasting glucose” OR “impaired glucose tolerance” OR “prediabetes” OR “diabetes” OR “lipids” OR “triglycerides” OR “cholesterol” OR “hypertriglyceridemia” OR “hyperlipidemia” OR “dyslipidemia” OR “CRP” OR “selectin” OR “interleukin” OR “anti-inflammatory” OR “inflammation” OR “oxidized LDL” OR “oxidative stress” OR “appetite” OR “satiety” OR “hunger” OR “overweight” OR “obesity”)). The language filter “English” was applied. The database search, which was performed by three reviewers (L.D., Z.H., S.D.), was finished on 1 July 2022. Furthermore, the reference lists of the included studies were perused to identify further studies of relevance.

### 2.2. Inclusion and Exclusion Criteria

This review considered only RCTs which investigated (1) the effect of an EC intake; (2) parameters of cardiovascular health (e.g., systolic blood pressure (SBP), diastolic blood pressure (DBP), FMD), glucose metabolism (e.g., glucose, insulin), lipid metabolism (e.g., TGs, LDL-C, HDL-C), inflammation (e.g., CRP, interleukins), oxidative stress (e.g., oxidized low-density lipoprotein (oxLDL)), as well as appetite sensations (e.g., satiety) and BW as outcome; (3) healthy participants, participants with overweight or obesity, and/or with cardiometabolic disorders. The latter included (pre)hypertension, hypertriglyceridemia, dysglycemia, (pre)diabetes, impaired endothelial function, atherosclerosis, and coronary heart disease.

Records were excluded for the following reasons: (1) no human intervention study (e.g., in vitro or animal study, review); (2) intervention with other flavonoids than EC (e.g., (+)-epicatechin, quercetin) or providing additional flavonoids or compounds beyond EC (e.g., by cocoa, dark chocolate, or green tea) unless these were also part of the control treatment; (3) EC not administered orally; (4) study without randomized controlled design; (5) no parameters of cardiometabolic outcomes (e.g., gene expression profiles); (6) subjects in particular life conditions (e.g., pregnancy) or suffering from severe diseases (e.g., cancer), or using medications which might favor cardiometabolic disorders (e.g., glucocorticoids, antiretroviral therapy).

### 2.3. Study Selection, Data Extraction, and Risk of Bias Assessment

Relevant studies were identified by checking the records for predefined eligibility criteria in the above-mentioned order. For this purpose, a self-made Excel template was used by three independent reviewers (L.D., Z.H., S.D.). First, all items were screened by title and/or abstract to exclude contributions that met exclusion criteria. Afterwards, all remaining studies were assessed for eligibility by reading the full-text article. Any discrepancies in the selection process were discussed until a consensus was reached. Finally, all studies which were considered to be eligible were included in this systematic review.

Afterwards, relevant data were extracted independently by L.D., Z.H., and S.D. by using a self-made Excel template: study design, details on intervention (amount, duration, and application of EC) and of the control/placebo treatment, participants (sample size, dropout rate, demographics, criteria of eligibility), sample size estimation, statistical analysis, and country in which the study had been performed. Further data extracted: outcomes (parameters of vascular function, glucose and lipid metabolism, inflammation, oxidative stress, appetite sensations, BW, and body composition), EC bioavailability, time points of investigations, conditions to ensure standardization (e.g., nutritional limitations, lifestyle instructions) and parameters/measures for verification (e.g., food records), compliance with intervention, and drop-outs considered. If available, the study registration was checked to complete data of relevance. With regard to the results, the average treatment effect, i.e., the difference between the changes between EC and control/placebo treatment, was considered to be relevant for each outcome. If the treatment effect was not provided in the original contribution, it was calculated by using available data (e.g., means ± SDs from pre- and postconsumption values of each treatment group). For this, a correlation between pre- and postconsumption data was assumed based on correlations between pre- and postintervention data of the placebo group (*n* = 48) of an own study [20]. Extracted were the direction of the treatment effect (significant increase or decrease), the lack of significant effects, and the corresponding *p*-values. Discrepancies in data extraction were also discussed by the authors. The effect of an EC intake on parameters of cardiometabolic health was estimated by a narrative synthesis based on the results from individual studies with respect to the target population, the type and content of intervention, the type of outcome, and the treatment effect.

Two review authors (L.D., S.D.) independently assessed the RoB of the included studies by applying the revised Cochrane Risk of Bias 2 (RoB 2) tool for randomized trials [21]. The RoB was judged for each outcome with regard to the following bias domains (D): (D1) bias arising from the randomization process, (D2) bias due to deviations from intended interventions, (D3) bias due to missing outcome data, (D4) bias in measurement of the outcome, and (D5) bias in selection of the reported result. Finally, the overall RoB was judged according to the approach of Sterne et al. [21], taking into account the RoB of each bias domain. Again, a self-made Excel template was used to check the results of each bias domain for each study. Discrepancies in the RoB assessment were also resolved through discussion with a third reviewer (S.E.). In addition, further factors that might act as confounder (e.g., changes in nutrition status or physical activity, low compliance with intervention) were considered.

## 3. Results

### 3.1. Study Selection and Study Characteristics

**Figure 1:**
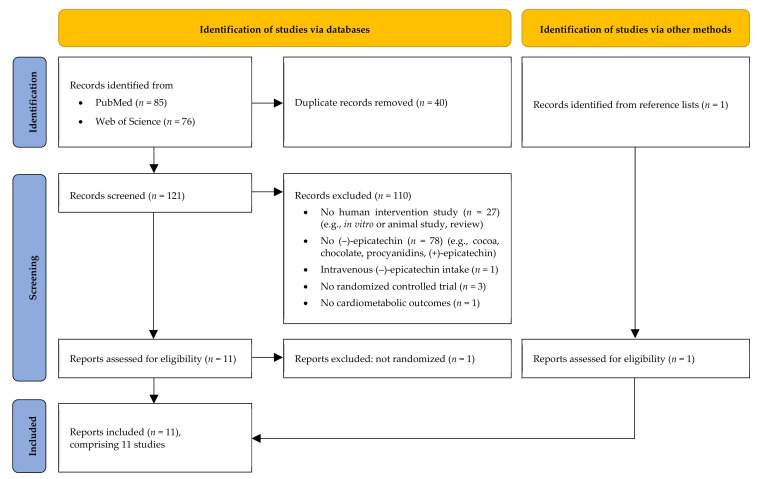
Flow diagram of study selection process modified according to the Preferred Reporting Items for Systematic Reviews and Meta-Analyses (PRISMA) 2020 statement [19].

After completing systematic literature search, 85 records were retrieved from PubMed and 76 from the Web of Science database, respectively. After duplicates had been removed, 121 records still remained. Based on titles and/or abstracts, 110 records were excluded (reasons: no human intervention study, *n* = 27; no isolated EC, *n* = 78; EC not administered orally, *n* = 1; no randomized controlled study design, *n* = 3; no cardiometabolic outcomes, *n* = 1). The full texts of the remaining 11 records were checked and a further publication was excluded due to a lack of randomized controlled study design (*n* = 1). Thereafter, a further suitable publication was found in the reference lists of the included records. Finally, 11 original contributions were considered to be eligible and were thus included in this review [20,22,23,24,25,26,27,28,29,30,31]. However, three of them present the results on different outcomes obtained from a single study [28,29,31]. Moreover, two out of eleven publications describe two studies each [25,28]. Hence, the results of 11 different RCTs are presented. A flow diagram of the identification and the selection of the studies is shown in Figure 1.

These trials were conducted in Germany [20,22], Australia [23,26], the Netherlands [24,28,29,31], USA [25], India [30], and in the UK [27]. Most RCTs were crossover studies [20,22,23,24,26,27,28,29,31], and only two studies had a parallel group design [25,30]. Seven studies investigated the effect of a bolus intake of EC on cardiometabolic parameters [22,23,24,25,26,27]. Three RCTs investigated regular [20,28,29,30,31] and one RCT the acute-on-chronic [28] EC intake. EC was provided in capsules [20,28,29,30,31], dissolved in water with [26] and without maltodextrin [22,23,27], or as part of a water-based drink prepared from alkalized and nonalkalized cocoa [25]. In one case, EC was encapsulated and ingested together with white chocolate [24]. In bolus studies, the applied dose of EC ranged from 0.1 to 2.0 mg/kg BW [22,25,27] and from 100 to 200 mg [23,24,26], respectively. RCTs investigating regular intake for 2 wk [20] and 4 wk [28,29,30,31] provided 25 mg/d [20] and 100 mg/d [28,29,30,31] EC, respectively. Participants were only men [22,23,24,25,27] or men and women [20,26,28,29,31]; Gutiérrez-Salmeán et al. [30] did not provide any information on gender. Subjects were healthy [22,23,24,25,27,28,29,31], healthy with only impaired endothelial function [26], or they were suffering from hypertriglyceridemia [30] or from a couple of risk factors of CVD [20]. Outcomes were parameters on vascular function [20,22,23,24,26,27,28,30], glucose [20,28,30,31] and lipid [20,28,30] metabolism, inflammation [29,30], oxidative stress [20,23], appetite sensations, food intake [25], BW [20,28,30], and body composition [20]. Plasma EC metabolites were partly assessed [20,23,24,26,27,28].

### 3.2. Effects of (–)-Epicatechin on Cardiometabolic Biomarkers

#### 3.2.1. Vascular Function

##### Acute Studies

Schroeter et al. [22] investigated the acute effects of pure EC in healthy men with normal weight (age: 25–32 y, BMI: 19–23 kg/m^2^) on FMD and peripheral arterial tonometry (PAT) index after an overnight fast. On different study days, subjects ingested 1 mg (*n* = 3) or 2 mg (*n* = 3) EC per kg BW dissolved in water or pure water as control (*n* = 6).

The FMD and PAT index increased 1 and 2 h after an intake of 1 mg EC/kg BW vs. control (*p* < 0.05), but not after 3 and 4 h (*p* ≥ 0.05). The intake of 2 mg EC/kg BW also increased the FMD and PAT index 2 h after EC compared to control (*p* < 0.05).

Alañón et al. [27] also examined the effects of pure EC intake on the FMD in healthy young men (*n* = 20, age: 23 ± 6 y, BMI: 23.2 ± 6.8 kg/m^2^; means ± SDs) after an overnight fast. In addition, peripheral SBP and DBP, laser Doppler imaging (LDI), plasma concentrations of nitrite and nitrate, as well as species derived from nitric oxide (NO_x_) were assessed. EC was administered in water and pure water was given as control. As by Schroeter et al. [22], a dose of 1.0 mg EC/kg BW was given, but lower amounts (0.1 mg EC/kg BW and 0.5 mg EC/kg BW) were also provided. FMD increased 2 h after ingestion of 0.5 mg EC/kg BW vs. control (*p* < 0.01). The administration of 1 mg EC/kg BW caused also an increase in FMD already 1 h (*p* < 0.01) and 2 h (*p* < 0.001) afterwards. No effects were observed at other time points of the investigation (0.5 mg kg/BW: 1 h, 4 h, 6 h; 1.0 mg kg/BW: 4 h, 6 h; all *p* ≥ 0.05) or after an intake of 0.1 mg EC/kg BW (1 h, 2 h, 4 h, 6 h; all *p* ≥ 0.05). Changes in further vascular parameters were not observed.

Loke et al. [23] provided pure EC (200 mg) dissolved in water compared to the EC-free control (water only) to healthy men (age: 43 ± 15 y, BMI: 25.1 ± 2.8 kg/m^2^; means ± SDs). In contrast to Schroeter et al. [22] and Alañón et al. [27], test drinks were given after breakfast (postprandial). Vascular metabolites (endothelin-1 (ET-1), *S*-nitrosothiols (SNO), nitrite) were assessed in plasma and urine. EC intake increased 2 h plasma concentrations of nitrite (*p* < 0.001) and SNO (*p* < 0.05) compared to control, whereas ET-1 in 2 h plasma decreased (*p* < 0.05). In 5 h total urine, nitrate increased after EC-enriched water had been administered compared to control (*p* < 0.05). The excretion of nitrite and ET-1 in 5 h urine and concentrations of nitrate in 2 h plasma were not affected by EC treatment (all *p* ≥ 0.05).

Ward et al. [26] also examined the effects of a postprandial EC intake on vascular function. Healthy men and women of older age (60 ± 8 y, mean ± SD) with impaired endothelial function (FMD 5.4 ± 1.2%, mean ± SD) ingested an EC-enriched drink (200 mg EC + 1 g maltodextrin, 200 mL water) and an EC-free control (1 g maltodextrin, 200 mL water) 2 h after a standardized breakfast. EC enrichment did not modulate FMD (after 1 h and 4 h, both *p* ≥ 0.05), SBP and DBP (after 0.5 h, 1 h, 1.5 h, 2 h, 2.5 h, 3 h, 3.5 h, and 4 h; all *p* ≥ 0.05), or plasma nitrite concentrations (after 1 h and 4 h, both *p* ≥ 0.05).

Dower et al. [24] investigated healthy men in advanced age (age: 62 ± 9 y, BMI: 25.1 ± 2.1 kg/m^2^; means ± SDs). On two different study days, they received 100 mg encapsulated EC and a placebo capsule, respectively, each together with 75 g white chocolate after an overnight fast. The acute supplementation of EC did not affect FMD, augmentation index for a heart rate of 75 bpm (AIx_75_), subendocardial viability ratio (SEVR), ejection duration, clinical SBP and DBP, endothelium-independent vasodilatation (EID), and plasma concentrations of NO_x_ and ET-1 vs. control treatment (all *p* ≥ 0.05).

An overview on studies that investigated the effect of an acute EC intake on vascular parameters is provided in Table 1.

##### Long-Term Studies

Dower et al. [28] examined the effects of a regular EC intake (100 mg/d for 4 wk) in healthy men and women, aged 66 ± 8 y (mean ± SD), compared to placebo. However, neither effects on FMD, pulse wave velocity (PWV), AIx_75_, SEVR, SBP and DBP (central BP, clinical BP, 24 h ambulatory BP), nor on NO_x_ and ET-1 concentrations in plasma were observed (all *p* ≥ 0.05).

Gutiérrez-Salmeán et al. [30] also investigated the chronic effects of a 4 wk intake of 100 mg EC per day in hypertriglyceridemic adults of both gender (age: 18–55 y; TGs: 3.2 ± 1.1 mmol/L, mean ± SD). Treatment with statins was only allowed if using a stable dose for at least 6 wk prior screening. A treatment effect on SBP and DBP was not detectable (both *p* ≥ 0.05).

Kirch et al. [20] examined adults with an increased CVD risk (age: 36 ± 14 y, BMI: 32.8 ± 5.6 kg/m^2^, means ± SDs, for further details see Table 2) who were not treated with antihypertensive, lipid-, and glucose-lowering drugs. Subjects received 25 mg EC per day for only 2 wk, but this did not affect SBP and DBP versus the placebo (both *p* ≥ 0.05).

Studies examining the effect of a regular EC intake on vascular function are shown in Table 2.

##### Acute-on-Chronic Study

After completion of the 4 wk EC treatment by Dower et al. [28], an additional dose of 100 mg EC was ingested after an overnight fast and the results were compared to the placebo treatment (Table 3). However, 2 h after this acute-on-chronic intake, FMD, EID, as well as NO_x_ concentration in plasma remained unchanged (all *p* ≥ 0.05).

#### 3.2.2. Glucose and Lipid Metabolism

Three long-term studies examined whether EC might affect glucose and lipid metabolism [20,28,30]. Further results of the study of Dower et al. [28] on glucose metabolism were published by van den Eynde et al. [31]. An overview is provided in Table 2.

Kirch et al. [20] did not observe any effects on glucose metabolism (FPG, insulin, HOMA-IR; all *p* ≥ 0.05) after 2 wk daily treatment with 25 mg EC vs. placebo. An intake of 100 mg EC daily for 4 wk reduced insulin (−1.46 mU/L; *p* = 0.03) and HOMA-IR in the study of Dower et al. [28] (−0.38; *p* = 0.04), whereas FPG remained unchanged (*p* ≥ 0.05). Differences in both FPG and insulin were not detectable due to EC treatment by Gutiérrez-Salmeán et al. [30] (both *p* ≥ 0.05). Van den Eynde et al. [31] assessed plasma dicarbonyls and advanced glycation end products (AGEs; free and protein-bound), but no effects on methylglyoxal (MGO), glyoxal (GO), 3-deoxyglucosone (3-DG), as well as on free and protein-bound N(ε)-(1-carboxyethyl)-lysine (CEL), N(ε)-(carboxymethyl)lysine (CML), N(δ)-(5-hydro-5-methyl-4-imidazolon-2-yl)-ornithine (MG-H1), and pentosidine could be observed (all *p* ≥ 0.05).

None of these studies observed any effects on TGs, TC, LDL-C, and HDL-C after a regular EC intake compared to placebo (all *p* ≥ 0.05) [20,28,30].

#### 3.2.3. Inflammation

Two of the 4 wk long-term studies [29,30] also determined biomarkers of inflammation (Table 2).

The daily intake of 100 mg EC decreased soluble E-selectin (sE-selectin; −7.7 ng/mL, *p* = 0.03) in plasma compared to placebo, whereas CRP, soluble intercellular adhesion molecule-1 (sICAM-1), soluble vascular adhesion molecule-1 (sVCAM-1), von Willebrand factor (vWf), monocyte chemotactic protein-1 (MCP-1), amyloid A (SAA), tumor necrosis factor alpha (TNF-α), interleukin-1beta (IL-1β), interleukin-6 (IL-6), and interleukin-8 (IL-8) in serum/plasma remained unchanged (all *p* ≥ 0.05) [29]. Gutiérrez-Salmeán et al. [30] investigated high sensitive CRP (hsCRP), but the treatment effect could not be calculated, as data on the correlation coefficient were not available.

#### 3.2.4. Antioxidant Status and Oxidative Stress

Details on the antioxidative status in plasma and/or urine were assessed in several acute studies [23,24,26,27], in the acute-on-chronic trial [28], and in long-term interventions [20,28].

Bolus intake of 100 mg EC [24] and 200 mg EC [23,26] increased the sum of glucuronidated, sulfated, and methylated epicatechin metabolites in plasma (AUC_0–8__h_ [24]; 2 h value [23,24]; after 1 h and 4 h, analytical details not provided [26]), and in 5 h urine [23]. A dose-dependent increase in the sum of glucuronidated, sulfated, and methylated epicatechin metabolites in plasma was observed by Alañón et al. [27] 1 h, 2 h (both *p* < 0.001), and 4 h (*p* < 0.01) after an intake of 0.5 mg EC/kg BW vs. the control. A dose of 1.0 mg EC/kg BW increased these EC metabolites at each time point of investigation compared to the control (after 1 h, 2 h, 4 h: all *p* < 0.001; after 6 h: *p* < 0.01). No changes were observed for the lowest EC dose (0.1 mg EC/kg BW) after 1 h, 2 h, 4 h, and 6 h (all *p* ≥ 0.05), as well as 6 h after ingestion of 0.5 mg EC/kg BW vs. the control (*p* ≥ 0.05). In the study of Dower et al. [28], EC was not detectable in fasting plasma before and after a 4 wk chronic EC intake; EC was only detectable in plasma 2 h after an acute-on-chronic EC supplementation (1950 ± 2070 nmol/L, mean ± SD). Before each intervention, EC concentrations of all 24 h urine samples were below the limit of detection (LOD: 300 nmol/L), whereas 51% of all samples reached concentrations above the LOD after 4 wk ingestion of 100 mg EC daily, but any sample after placebo treatment. In the study of Kirch et al. [20], the sum of glucuronidated and sulfated EC metabolites in fasting plasma was quantifiable in 15% of the subjects after EC treatment (25 mg/d, 2 wk), reaching concentrations between 13.4 and 277.9 nmol/L; 79% had levels below the limit of quantification (8.6 nmol/L) and 6% below the LOD (<5.2 nmol/L).

After daily treatment with 25 mg EC in the study of Kirch et al. [20], the concentrations of vitamin C, E, and β-carotene in plasma, as well as the vitamin-E-to-cholesterol ratio remained unchanged vs. the placebo (all *p* ≥ 0.05).

The effects of an acute and regular EC ingestion on parameters of oxidative stress were determined by Loke et al. [23] (Table 1) and Kirch et al. [20] (Table 2), respectively. After a single EC intake (200 mg) vs. EC-free placebo, F_2_-isoprostanes (F_2_-isoPS) in 2 h plasma and 5 h urine remained unchanged (both *p* ≥ 0.05) [23]. In the study of Kirch et al. [20], a 2 wk intake of 25 mg EC per day did neither modulate oxLDL concentration in plasma nor the ratio of oxLDL to LDL-C compared to the placebo treatment (all *p* ≥ 0.05).

#### 3.2.5. Appetite Sensations, Food Intake and Body Weight

Greenberg et al. [25] (Table 1) examined the effects of an acute EC ingestion on appetite sensations in healthy men (*n* = 28, age: 23 ± 4 y; BMI: 23.3 ± 2.4 kg/m^2^; means ± SDs). In their crossover study, subjects consumed an alkalized cocoa drink enriched with EC (1 mg/kg BW) and without EC enrichment (control; 0 mg EC) on different study days. The AUCs_0–2.5__h_ for appetite sensations showed an increase in hunger (*p* < 0.0001) and emptiness (*p* < 0.0001) by EC treatment, whereas satiety (*p* = 0.0002) and fullness (*p* < 0.0001) were reduced. However, 2.5 h after ingestion of the EC-containing drink, no effect on *ad libitum* food intake (amount of pizza consumed) was observed compared to control (*p* ≥ 0.05). In a further RCT of Greenberg et al. [25], which was performed in a parallel design, a subgroup (*n* = 14) consumed a single nonalkalized cocoa drink containing 0.6 mg EC/kg BW either enriched with 1.0 g EC (intervention drink: 1.6 mg EC/kg BW; *n* = 7) or not (control drink: 0.6 mg EC/kg BW; *n* = 7). However, *ad libitum* food intake after 2.5 h was not modulated by the EC treatment (*p* ≥ 0.05).

In all long-term studies, EC intake modulated neither BW [20,28,29,31] (BW not measured [30]) nor FM and fat distribution [20].

**Table 1:** Effect of an acute (–)-epicatechin intake on cardiometabolic parameters—results from randomized controlled trials.

Study (Country)	Design	*n* ^1^	Participants ^2^	Intervention	Results ^3^	Details
Schroeter et al. [22] (Germany)	RCT investigator-blind, crossover	6	Healthy men Exclusion criteria: diabetes, hypertension, hypercholesterolemia, acute inflammation, use of dietary supplements Gender (m/f): 6/0 Age: 25−32 y BMI: 19−23 kg/m²	**I_1_ (*n* = 3):** 1 mg EC/kg BW dissolved in 3 mL/kg BW of water **I_2_ (*n* = 3):** 2 mg EC/kg BW dissolved in 3 mL/kg BW of water **C (*n* = 6):** water only (3 mL/kg BW)	**Vascular Parameters** ↑ FMD (**I_1_**: t_1 h_, t_2 h_; **I_2_**: t_2 h_), PAT index (**I_1_**: t_1 h_, t_2 h_; **I_2_**: t_2 h_) ↔ FMD (**I_1_**: t_3 h_, t_4 h_)	Instructions: ο Overnight fast: ≥ 12 h
Loke et al. [23] (Australia)	RCT crossover, 1 wk washout	12	Healthy men Exclusion criteria: chronic diseases; > 20 g alcohol/d; use of medications, vitamin supplements or antioxidants Gender (m/f): 12/0 Age: 43 ± 15 y BMI: 25.1 ± 2.8 kg/m² SBP: 123 ± 7 mmHg DBP: 78 ± 7 mmHg TGs: 1.7 ± 1.6 mmol/L TC: 4.9 ± 1.0 mmol/L LDL-C: 2.7 ± 1.1 mmol/L HDL-C: 1.5 ± 0.8 mmol/L	**I:** 200 mg EC dissolved in 300 mL of water **C:** water only (300 mL) **Setting:** postprandial	**Vascular Parameters** ↓ 2-h-plasma: ET-1 (*p* < 0.05) ↑ 2-h-plasma: SNO (*p* < 0.05), nitrite (*p* < 0.001); 5-h-total urine: nitrate (*p* < 0.05) ↔ 2-h-plasma: nitrate; 5-h-total urine: ET-1, nitrite **Other** ↔ 2-h-plasma: F_2_-isoPS; 5-h-total urine: F_2_-isoPS	Instructions: ο 48 h prior treatments: no flavonoid-rich foods (e.g., fruit juice, chocolate, cocoa, tea, red wine) ο 24 h prior treatments: no alcohol or vigorous physical activity ο Morning prior treatments: intake of a breakfast of the same composition Both treatments on the same weekday and time point
Dower et al. [24] (The Netherlands)	RCT double-blind, crossover, 2 wk washout	20	Healthy male non-smokers (age: 40–80 y, BMI: >20 and ≤30 kg/m²) Exclusion criteria ^4^: diabetes mellitus; cardiovascular, gastrointestinal, or liver diseases; antihypertensive or cholesterol-lowering medication; adherence to a prescribed diet; unstable weight in the last 2 months; moderate physical activity (>10 h/wk) Gender (m/f): 20/0 Age: 62 ± 9 y BMI: 25.1 ± 2.1 kg/m² SBP: 122 ± 15 mmHg DBP: 74 ± 6 mmHg FMD: 3.0 ± 1.6 % AIx_75_: 19.3 ± 11.9 %	**I:** 100 mg EC (2 × 50 mg, 2 capsules) + 75 g white chocolate **C:** placebo (2 capsules) + 75 g white chocolate	**Vascular Parameters** ↔ FMD (t_2 h_), AIx_75_ (t_2 h_), SEVR (t_2 h_), ejection duration (t_2 h_), SBP (t_2 h_), DBP (t_2 h_), NO_x_ (t_2 h_), ET-1 (t_2 h_)	Instructions: ο 1 wk prior and throughout the study period: no flavonoid-rich foods (e.g., cocoa products, red wine, apples); limit coffee and tea (≤1 cup/d) ο 24 h prior treatments: no alcohol or physical activity ο Evening prior treatments: intake of a provided standardized low-flavonoid dinner (overnight fast) ο Intake of a standardized flavonoid-free breakfast after completing vascular function measurements
Greenberg et al. [25] (USA)	RCT double-blind, crossover, 1 wk washout	28 (30)	Healthy male non-smokers (BMI: 18.5–30.0 kg/m²) Exclusion criteria ^4^: intake of coffee, tea, sodas containing caffeine (>1 serving/d) or alcohol (≥2 drinks/d); vigorous physical activity; appetite-affecting medications; allergy to chocolate, cocoa, or pizza; weight changes (≥ 5% in the past 6 months) Gender (m/f): 28/0 Age: 23 ± 4 y BMI: 23.3 ± 2.4 kg/m² WC: 81.4 ± 7.4 cm WHR: 0.81 ± 0.05	**I:** 1.0 mg EC/kg BW + alkalized, EC-free cocoa dissolved in 2.96 mL/kg BW of warm water **C:** alkalized, EC-free cocoa dissolved in 2.96 mL/kg BW of warm water	**Appetite Sensations** ↑ Hunger-AUC_0–2.5 h_ *(p* < 0.0001), emptiness-AUC_0–2.5 h_ (*p* < 0.0001) ↓ Satiety-AUC_0–2.5 h_ (*p* = 0.0002), fullness-AUC_0–2.5 h_ (*p* < 0.0001) ↔ Food intake (*ad libitum*, t_2.5 h_)	Instructions: ο Throughout the study period: no chocolate or cocoa beverages; tea, coffee, or other caffeinated drinks; tobacco or nicotine products ο 48 h prior treatments: no alcohol, psychotropic drugs or physical activity ο 24 h prior treatments: similar diet (food quantities and groups, amounts of macronutrients) ο During treatment: remain seated Compliance assessment: behavioral, health, and dietary-recall questionnaire
Greenberg et al. [25] (USA)	RCT double-blind, parallel	14	Subgroup of the crossover study (details see above)	**I:** 1.0 mg EC/kg BW + non-alkalized cocoa with 0.6 mg EC/kg BW dissolved in 2.96 mL/kg BW of warm water **C:** non-alkalized cocoa with 0.6 mg EC/kg BW dissolved in 2.96 mL/kg BW of warm water	**Appetite Sensations** ↔ Food intake (*ad libitum*, t_2.5 h_)	See above
Ward et al. [26] (Australia)	RCT double-blind, crossover, 1 wk washout	14 (16)	Healthy non-smokers with impaired endothelial function (peak FMD: 3–8%) Exclusion criteria: chronic diseases, SBP: < 100 or > 160 mmHg, DBP: < 50 or > 100 mmHg, elevated cholesterol, BMI: < 18 or > 35 kg/m², antihypertensive or cholesterol-lowering medications, recent weight changes (> 6% of BW), food allergies, pregnancy or lactation Gender (m/f): 6/10 Age: 60 ± 8 y BMI: 24.7 ± 3.3 kg/m² SBP: 115 ± 9 mmHg DBP: 68 ± 7 mmHg FMD: 5.4 ± 1.2 %	**I:** 200 mg EC + 1 g maltodextrin dissolved in 200 mL of warm water **C:** 1 g maltodextrin dissolved in 200 mL of warm water **Setting**: postprandial	**Vascular Parameters** ↔ FMD peak and continuous response (t_1 h_, t_4 h_), SBP and DBP (t_0.5 h_, t_1 h_, t_1.5 h_, t_2 h_, t_2.5 h_, t_3 h_, t_3.5 h_, t_4 h_), plasma nitrite (t_1 h_, t_4 h_)	Instructions: ο 24 h prior treatments: limit EC-rich foods (no red wine or dark chocolate, and ≤2 pieces of fruits) ο 12 h prior treatments: no tea and coffee ο Approx. 2 h prior treatments: intake of a standardized breakfast
Alañón et al. [27] (United Kingdom)	RCT double-blind, crossover, 2 wk washout	20	Healthy male non-smokers (age: 18–40 y, BMI: 20.0–27.5 kg/m²) Exclusion criteria ^4^: cardiovascular-related (e.g., hypertension, BP: ≥ 140/90 mmHg) or metabolic (diabetes) disorders; abnormal hematological parameters (liver enzymes, hemoglobin, hematocrit and leukocyte counts); antihypertensive, anti-inflammatory medication or antibiotics within 2 months prior trial; extreme exercise routine; vegetarian or vegan diet; use of nutritional supplements within 2 months prior trial Gender (m/f): 20/0 Age: 23 ± 6 y BMI: 23.2 ± 6.8 kg/m² SBP: 124 ± 5 mmHg DBP: 66 ± 5 mmHg TGs: 1.0 ± 0.4 mmol/L TC: 4.5 ± 0.7 mmol/L FMD: 5.9 ± 1.1 %	**I_1_:** 0.1 mg EC/kg BW dissolved in 3 mL/kg BW of low-nitrate water **I_2_:** 0.5 mg EC/kg BW dissolved in 3 mL/kg BW of low-nitrate water **I_3_:** 1.0 mg EC/kg BW dissolved in 3 mL/kg BW of low-nitrate water **C:** water only	**Vascular Parameters** ↑ FMD (**I_2_**: t_2 h_ [*p* < 0.01]; **I_3_**: t_1 h_ [*p* < 0.01], t_2 h_ [*p* < 0.001]) ↔ FMD (**I_1_**: t_1 h_, t_2 h_, t_4 h_, t_6 h_; **I_2_**: t_1 h_, t_4 h_, t_6 h_; **I_3_**: t_4 h_, t_6 h_); SBP and DBP (**I_1–3_**: t_1 h_, t_2 h_, t_4 h_, t_6 h_); LDI (**I_1–3_**: t_2 h_, t_4 h_, t_6 h_); plasma nitrite, nitrate and NO_x_ (**I_1–3_**: t_1 h_, t_2 h_, t_4 h_, t_6 h_)	Instructions: ο Throughout the study period: maintain lifestyle (normal diet, activity and fluid intake), but restrict vigorous exercise (< 20 min/d) and alcohol intake (< 168 g/wk) ο 24 h prior treatments: limit polyphenol-containing foods (e. g, fruits, vegetables, cocoa, tea, wine), avoid nitrate-rich foods/beverages (e.g., leafy green vegetables, beetroot, processed meat, tap water) ο Evening prior treatments: intake of a standardized, low-fat, low-polyphenol meal before 8 p.m. (overnight fast: ≥12 h)

^1^ number of participants completing the study (number of participants included), ^2^ baseline characteristics are expressed as means ± SDs or medians [IQRs]. SDs were calculated if these were not provided (SD = SEM × √*n*). Age and BMI are expressed in ranges if means ± SDs were not given, ^3^ average treatment effect; ↓ treatment effect with significant decrease (*p* ≤ 0.05), ↑ treatment effect with significant increase (*p* ≤ 0.05), ↔ treatment effect not detectable (*p* > 0.05); ^4^ only exclusion criteria from the original contribution were given; further criteria, more details and/or divergent information are provided in the respective study registration. AIx_75_, augmentation index corrected for a heart rate of 75 bpm; AUC, area under the curve; BMI, body mass index; BP, blood pressure; BW, body weight; C, control; DBP, diastolic blood pressure; EC, (–)-epicatechin; ET-1, endothelin-1; F_2_-isoPS, F_2_-isoprostane; f, female; FMD, flow-mediated dilatation; γ-GT, gamma-glutamyltransferase; HDL-C, high-density lipoprotein cholesterol; I, intervention; LDI, laser Doppler imaging; LDL-C, low-density lipoprotein cholesterol; m, male; NO_x_, species derived from nitric oxide; PAT index, peripheral arterial tonometry index; RCT, randomized controlled trial; SBP, systolic blood pressure; SEVR, subendocardial viability ratio; SNO, *S*-nitrosothiols; TC, total cholesterol; TGs, triglycerides; WC; waist circumference; WHR, waist-to-hip ratio.

**Table 2:** Effect of a regular (–)-epicatechin intake on cardiometabolic parameters—results from randomized controlled trials.

Study (Country)	Design	*n* ^1^	Participants ^2^	Intervention	Duration	Results ^3^	Details
Dower et al. [28] (The Netherlands)	RCT double-blind, crossover, 4 wk washout	35 (37)	Healthy non-smokers (age: 40–80 y, BMI: 20–40 kg/m², SBP: 125–160 mmHg) ^4^ Exclusion criteria ^4^: diabetes mellitus; cardiovascular, gastrointestinal, or liver diseases; antihypertensive or cholesterol-lowering medication or corticosteroids; adherence to a prescribed diet; unstable weight in the last 2 months; moderate to vigorous physical activity (≥10 h/wk, ≥3 metabolic equivalent tasks); pregnancy or lactation Gender (m/f): 25/12 Age: 66 ± 8 y BMI: 26.7 ± 3.3 kg/m² SBP: 129 ± 14 mmHg DBP: 75 ± 10 mmHg FPG: 5.7 ± 0.7 mmol/L Insulin: 6.1 ± 3.8 mU/L HOMA-IR: 1.6 ± 1.0 TGs: 1.3 ± 0.6 mmol/L TC: 5.6 ± 0.9 mmol/L LDL-C: 3.5 ± 0.8 mmol/L HDL-C: 1.5 ± 0.4 mmol/L PWV: 12.9 ± 1.9 m/s AIx_75_: 25.9 ± 7.6 %	**I:** 100 mg EC (2 × 50 mg; 2 capsules, 1 for breakfast and dinner each) **C:** placebo (2 capsules)	4 wk	**Vascular Parameters** ↔ FMD, PWV, AIx_75_, EID, SBP (office, 24 h, central), DBP (office, 24 h, central), SEVR, NO_x_, ET-1 **Glucose Metabolism** ↓ Insulin (*p* = 0.03), HOMA-IR (*p* = 0.04) ↔ FPG **Lipid Metabolism** ↔ TGs, TC, LDL-C, HDL-C **BW** ↔ BW	Instructions: ο 1 wk prior and throughout the study period: no EC-rich foods (e.g., cocoa-containing products, red wine and apples), limit coffee and tea (≤ 1 cup/d) ο 24 h prior study days: no alcohol intake or physical activity ο Evening prior study days: provided standardized low-flavonoid dinner (overnight fast)
Dower et al. [29] (The Netherlands)	For details (design, *n*, participants, intervention and duration), see Dower et al. [28]					**Inflammation** ↓ sE-selectin (*p* = 0.03) ↔ sICAM-1, sVCAM-1, MCP-1, vWf, CRP, IL-1β, IL-6, IL-8, SAA, TNF-α	See Dower et al. [28]
Gutiérrez-Salmeán et al. [30] (India)	RCT double-blind, parallel	30	Hypertriglyceridemic (age: 18–55 y, TGs: 5.2–13.0 mmol/L) without pharmacologic treatment or with a stable dose of statins ≥ 6 wk prior to screening Exclusion criteria: high cardiovascular risk, arterial hypertension (SBP ≥ 140 and/or DBP ≥ 90 mmHg), stroke, transient ischemic attack, unstable cardiac disease, abnormal ECG, pancreatitis, uncontrolled diabetes (HbA1c > 9% and/or fasting glycemia > 200 mg/dL), hypoglycemia, renal failure (GFR < 60 mL/min), HIV, hepatitis B or C infection or coagulopathy; ≥ 14 alcoholic drinks/wk; use of insulin, anticoagulant, anti-platelet or anti-clotting therapy (e.g., daily aspirin, coumadin), atypical antipsychotics, beta-blockers, glucocorticoids, isotretinoin, and tamoxifene; participation in another clinical trial (≤ 30 d) Gender (m/f): n. a. Age: 18–55 y BMI: n. a. SBP: 122 ± 6 mmHg DBP: 81 ± 4 mmHg FPG: 8.3 ± 3.4 mmol/L Fructosamine: 314.3 ± 107.3 μmol/L TGs: 3.2 ± 1.1 mmol/L TC: 5.31 ± 12.6 mmol/L LDL-C: 3.5 ± 1.1 mmol/L HDL-C: 4.4 ± 1.1 mmol/L	**I (*n* = 20):** 100 mg EC/d (4 × 25 mg, 2 capsules each 30 min before lunch and dinner) **C (*n* = 10):** placebo capsules	4 wk	**Vascular Parameters ^5^** ↔ SBP, DBP **Glucose Metabolism ^6^** ↔ FPG, insulin **Lipid Metabolism ^7^** ↔ TGs, TGs/HDL-C, TC, LDL-C, HDL-C, non-HDL-C **Inflammation ^8^**	Instructions: ο Throughout the study period: “healthy” diet if possible, without known sources of flavanols (e.g., berries, green tea)
van den Eynde et al. [31] (The Netherlands)	For details (design, *n*, participants, intervention and duration), see Dower et al. [28]					**Glucose Metabolism** ↔ Plasma dicarbonyls (MGO, GO, 3-DG); plasma AGEs (free and/or protein-bound CML, CEL, MG-H1, pentosidine)	See Dower et al. [28]
Kirch et al. [20] (Germany)	RCT double-blind, crossover, 2 wk washout	47 (48)	Overweight or obese (BMI: ≥ 25 kg/m²) non-smokers at cardiovascular risk (SBP ≥ 130 or DBP ≥ 85 mmHg, and FPG > 5.55 mmol/L or fasting TGs > 1.69 mmol/L or TC > 5.2 mmol/L) Exclusion criteria: chronic diseases (e.g., cardiovascular, hepatic, renal, pulmonary), intake of antihypertensive/glucose- or cholesterol-lowering medications, regular use of nutritional supplements, planned weight reduction, drug or alcohol dependency, pregnancy or lactation Gender (m/f): 25/22 Age: 36 ± 14 y BMI: 32.8 ± 5.6 kg/m² SBP: 135 ± 12 mmHg DBP: 89 ± 9 mmHg FPG: 5.8 ± 0.6 mmol/L Insulin: 12.5 ± 6.8 mU/L HOMA-IR: 3.3 ± 1.9 TGs: 2.0 ± 1.1 mmol/L TC: 5.9 ± 1.0 mmol/L LDL-C: 3.7 ± 0.8 mmol/L HDL-C: 1.3 ± 0.3 mmol/L	**I:** 25 mg EC/d (capsule) **C:** placebo (capsule)	2 wk	**Vascular Parameters** ↔ SBP, DBP **Glucose Metabolism** ↔ FPG, insulin, HOMA-IR **Lipid Metabolism** ↔ TGs, TC, LDL-C, HDL-C **Oxidative Status/Stress** ↔ Vitamin C, vitamin E, β-carotene, vitamin E/TC, oxLDL, oxLDL/LDL-C **BW/Body Composition** ↔ BW, BMI, FM, waist circumference, WHR	Instructions: ο 3 d prior and throughout the study period: normal diet, but restricted for EC-rich food (green and black tea: ≤ 2 cups/d, fruit juice and sweetened fruit juice blend: ≤ 1 glass/d; apples and pears: ≤ 2 pieces/wk) and physical activity ο Overnight fast: 12 h Compliance assessments: ο 3-d food record prior study days to check diet behavior ο Diary for capsule intake and returned blister packages to check compliance with intervention

^1^ number of participants completing the study (number of participants included), ^2^ baseline characteristics are expressed as means ± SDs or medians [IQRs]. SDs were calculated if these were not provided (SD = SEM × √*n*) and were calculated for all participants if given separately for males/females or intervention/placebo group. Age and BMI are expressed in ranges if means ± SDs were not given. ^3^ average treatment effect if not indicated otherwise; ↓ treatment effect with significant decrease (*p* ≤ 0.05), ↑ treatment effect with significant increase (*p* ≤ 0.05), ↔ treatment effect not detectable (*p* > 0.05); ^4^ only inclusion and exclusion criteria from the original contribution were given; further criteria, more details and/or divergent information are provided in the respective study registration. ^5^ treatment effect was calculated using *r* = 0.508 for SBP and *r* = 0.539 for DBP [20]; ^6^ treatment effect was calculated considering *r* = 0.840 for glucose and *r* = 0.393 for insulin; not possible for fructosamine as *r* was not available [20], ^7^ treatment effect was calculated using *r* = 0.780 for TGs, *r* = 0.825 for TC, *r* = 0.852 for LDL-C, *r* = 0.881 for HDL-C, *r* = 0.783 for TG/HDL-C, *r* = 0.875 for non-HDL-C [20]; ^8^ hsCRP was determined, but the treatment effect could not be calculated as *r* was not available [20]. 3-DG, 3-deoxyglucosone; AGEs, advanced glycation end products; AIx_75_, augmentation index corrected for a heart rate of 75 bpm; BMI, body mass index; C, control; CEL, N(ε)-(1-carboxyethyl)lysine; CML, N(ε)-(carboxymethyl)lysine; CRP, c-reactive protein; DBP, diastolic blood pressure; EC, (–)-epicatechin; ECG, electrocardiogram; EID, endothelium-independent dilatation; ET-1, endothelin-1; f, female; FM, fat mass; FMD, flow-mediated dilatation; FPG, fasting plasma glucose; GFR, glomerular filtration rate; GO, glyoxal; HDL-C, high-density lipoprotein cholesterol; HOMA-β, homeostatic model assessment of beta cell function; HOMA-IR, homeostatic model assessment of insulin resistance; hsCRP, high sensitive c-reactive protein; I, intervention; IL-1β, interleukin-1beta; IL-6, interleukin-6; IL-8, interleukin-8; LDL-C, low-density lipoprotein cholesterol; m, male; MCP-1, monocyte chemotactic protein-1; MG-H1, N(δ)-(5-hydro-5-methyl-4-imidazolon-2-yl)-ornithine; MGO, methylglyoxal; NO_x_, species derived from nitric oxide; oxLDL, oxidized low-density lipoprotein; PWV, pulse wave velocity; RCT, randomized controlled trial; SAA, serum amyloid A; SBP, systolic blood pressure; sE-selectin, soluble endothelial selectin; SEVR, subendocardial viability ratio; sICAM-1, soluble intercellular adhesion molecule-1; sVCAM-1, soluble vascular adhesion molecule-1; TC, total cholesterol; TGs, triglycerides; TNF-α, tumor necrosis factor alpha; vWf, von Willebrand factor; WHR, waist-to-hip ratio.

**Table 3:** Effect of an acute (–)-epicatechin intake following a regular ingestion of (–)-epicatechin on cardiometabolic parameters—results from randomized controlled trials.

Study (Country)	Design	*n* ^1^	Participants	Intervention	Results ^2^	Details
Dower et al. [28] (The Netherlands)	RCT double-blind, crossover, 4 wk washout	35 (37)	See Table 2	***Acute-on-chronic:*** *at the end of the long-term treatment (4 wk,*Table 2*), subjects received an* *additional daily dose (see below)* **I:** 100 mg EC (2 capsules) **C:** placebo (2 capsules)	**Vascular Parameters** ↔ FMD (t_2h_), EID (t_2h_), NO_x_ (t_2h_)	See Table 2

^1^ number of participants completing the study (number of participants included), ^2^ average treatment effect; based on values determined in fasting state before starting 4 wk intervention (baseline) and values obtained 2 h after an acute intake after completing the 4 wk intervention; ↔ treatment effect not detectable (*p* > 0.05). EC, (–)-epicatechin; EID, endothelium-independent dilatation; f, female; FMD, flow-mediated dilatation; NO_x_, species derived from nitric oxide; RCT, randomized controlled trial.

### 3.3. Risk of Bias Assessment

**Figure 2:**
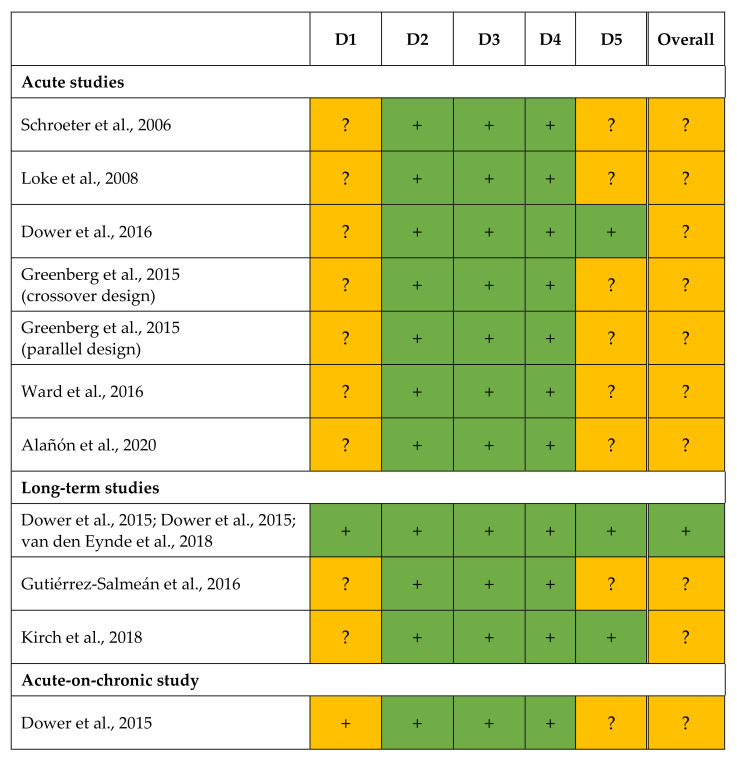
Results of the risk of bias assessment according to the revised Cochrane risk of bias 2 tool for randomized trials [21]. + low risk, ? some concerns, – high risk. D1, bias arising from the randomization process; D2, bias due to deviations from intended interventions; D3, bias due to missing outcome data; D4, bias in measurement of the outcome; D5, bias in selection of reported result [20,22,23,24,25,26,27,28,29,30,31].

RoB was assessed for each outcome based on qualitative criteria (Table 4). The judgements are shown in Figure 2.

All trials had a randomized controlled study design [20,22,23,24,25,26,27,28,29,30,31], but the methodical details on randomization were not described in two publications [22,25]. The order of assignment was obscured in the RCT of Dower et al. [28,29,31] until participants were enrolled and assigned to interventions; this remains unclear for further studies [20,22,23,24,25,26,27,30]. As differences at baseline between intervention and placebo group [30] or between EC and control treatments [20,22,23,24,25,26,27,28,29] were not observed in any study, problems with the randomization process are unlikely. Thus, bias arising from the randomization process (D1) was assessed with either low risk [28,29,31] or with some concern [20,22,23,24,25,26,27,30] for all outcomes.

In almost all trials, participants [20,24,25,26,27,28,29,30,31] and/or investigators [20,22,24,25,26,27,28,29,30,31] were blinded to treatment. In the RCTs of Schroeter et al. [22] and Loke et al. [23], it remains unclear whether subjects [22,23] and/or investigators [23] were aware of the assigned intervention during trial. In both publications, however, no deviations from the intended interventions arose due to the trial context. Furthermore, all studies used an appropriate statistical analysis (intention-to-treat analyses or modified intention-to-treat analyses excluding participants with missing outcome data) [20,22,23,24,25,26,27,28,29,30,31]. Accordingly, the bias due to deviations from the intended interventions (D2) was always judged with low risk [20,22,23,24,25,26,27,28,29,30,31].

For each endpoint, data for all [20,22,23,24,27,30] or nearly all [25,26,28,29,31] participants randomized were available. Dower et al. (long-term study) [28,29,31], Ward et al. [26], and Greenberg et al. [25] reported dropouts, but there were no hints that the results were biased by missing outcome data. Therefore, the bias due to missing outcome data (D3) was always rated with low risk [20,22,23,24,25,26,27,28,29,30,31]. The methods used to measure the outcomes were always appropriate and no methodological differences were reported within a study between the treatments [20,22,23,24,25,26,27,28,29,31] or groups [25,30]. In most studies, outcome assessors were blinded to all outcomes [22,24,26,27,28,29,30,31]. The data analysts of Kirch et al. [20] were aware of the intervention when analyzing plasma EC concentrations, but they were blinded for further data until statistical analysis was completed. Loke et al. [23] provided no information whether statistical analysis was done in blinded manner; Greenberg et al. [25] stated that the outcome assessor was not blinded to intervention while evaluating the results. However, there is no indication that this has affected the results [20,23,25]. Correspondingly, the RoB in measurement (D4) was judged as low for all outcomes [20,22,23,24,25,26,27,28,29,30,31].

If information about study registration was missing, bias in selection of the reported result (D5) was always assessed with some concerns [22,23,26,30]. The same judgement was done for the substudies of Dower et al. [28] (acute-on-chronic study) and Greenberg et al. [25] (parallel group study), as these were not mentioned in the registration of the main study. However, study registration was available for most studies [20,24,25,27,28,29,31]. In the acute and long-term study of Dower et al. [24,28,29,31] and in the trial of Kirch et al. [20], data analyses followed a prespecified plan that had been finalized before unblinded outcome data were available (except for EC plasma concentration [20]). In addition, a selection of the presented results from multiple eligible outcome measurements (e.g., time points) or from multiple eligible analysis of the data is unlikely. Therefore, the RoB in D5 was rated as low for all endpoints [28,29,31]. As Greenberg et al. (crossover design) [25] were not blinded while analyzing data, and no information was available on the result selection, the outcomes were judged with some concerns. The results of Alañón et al. [27] were also assessed with some concerns in D5 because blinding during data analysis remained unclear and the possibility of data selection could not be ruled out.

Due to some concerns in D1 [20,22,23,24,25,26,27,28,30] and/or D5 [22,23,25,26,27,28,30], the overall RoB was judged with some concerns for the outcomes of Schroeter et al. [22], Loke et al. [23], Dower et al. (acute study) [24], Ward et al. [26], Greenberg et al. [25] (crossover and parallel study), Alañón et al. [27], Gutiérrez-Salmeán et al. [30], Kirch et al. [20], and Dower et al. [28] (acute-on-chronic study). The long-term RCT of Dower et al. [28,29,31] showed a low RoB for each domain. Thus, the overall RoB for their endpoints was assessed as low.

**Table 4:** Criteria on studies’ quality to assess the risk of bias.

	Acute Studies							Long-Term Studies			Acute-on-Chronic Study
	Schroeter et al. [22]	Loke et al. [23]	Dower et al. [24]	Greenberg et al. [25] (crossover design)	Greenberg et al. [25] (parallel design)	Ward et al. [26]	Alañón et al. [27]	Dower et al. [28]; Dower et al. [29]; van den Eynde et al. [31]	Gutiérrez-Salmeán et al. [30]	Kirch et al. [20]	Dower et al. [28]
**Study design**											
Controlled	✓	✓	✓	✓	✓	✓	✓	✓	✓	✓	✓
Crossover	✓	✓	✓	✓	✕	✓	✓	✓	✕	✓	✓
Parallel group	✕	✕	✕	✕	✓	✕	✕	✕	✓	✕	✕
Randomized	✓	✓	✓	✓	✓	✓	✓	✓	✓	✓	✓
List generated before study started	?	✓	✓	?	?	✓	✓	✓	✓	✓	✓
Adequate randomization method	?	✓	✓	?	?	✓	✓	✓	✓	✓	✓
Allocation concealment	?	?	?	?	?	?	?	✓	?	?	✓
Blinded	✓	✓	✓	✓	✓	✓	✓	✓	✓	✓	✓
Participants	?	?	✓	✓	✓	✓	✓	✓	✓	✓	✓
Investigators	✓	?	✓	✓	✓	✓	✓	✓	✓	✓	✓
Outcome assessments	?	?	✓	✓	✓	✓	?	✓	?	✓^1^	✓
Prior registration of the study protocol	✕	✕	✓	✓	✕	✕	✓	✓	✕	✓	✕
**Methods**											
Details on intervention	✓	✓	✓	✓	✓	✓	✓	✓	✓	✓	✓
Details on the investigation of outcome markers	✓	✓	✓	✓	✓	✓	✓	✓	✓	✓	✓
Considering potential confounders											
Compliance	✕	✕	✕	✓	✓	✕	✕	✓	✕	✓	✕
Nutritional behavior	✕	(✓)	(✓)	✓	✓	(✓)	(✓)	(✓)	(✓)	✓	(✓)
Physical activity	✕	(✓)	(✓)	(✓)	(✓)	✕	(✓)	(✓)	✕	(✓)	(✓)
Body weight/body composition	✕/✕	✕/✕	✕/✕	✕/✕	✕/✕	✕/✕	✕/✕	✓/✕	✕/✕	✓/✓	✓/✕
Medication	✓	✓	✓	✓	✓	✓	✓	✓	✓	✓	✓
**Statistics**											
Sample size calculation performed	✕	✕	✓^2^	✕^3^	✕	✕	✓^2^	✓^2^	✓^4^	✓^5^	✕
Details on statistical analysis described	✓	✓	✓	✓	✓	✓	✓	✓	✓	✓	✓
Intention-to-treat analysis	✓	✓	✓	✓	✓	✓	✓	✓	✓	✓	✓
Per-protocol analysis	✕	✕	✕	✕	✕	✕	✕	✕	✕	✓	✕
**Results**											
Dropout, reasons reported	✕, N/R	✕, N/R	✕, N/R	✓, ✕	?	✓, ✓	✕, N/R	✓, ✓	✕, N/R	✓, ✓	✓, ✓
Outcomes reported according to registration	−	−	✓^6^	✓^7^	−	−	✓^8^	✓^9^	−	✓^10^	−

✓ yes; (✓) yes, but adherence with instructions not checked; ✕ no; ? unclear, no details given; − cannot be judged as study was not registered; N/R not relevant. ^1^ except for plasma (–)-epicatechin concentration, ^2^ sample size estimation for flow-mediated dilatation, ^3^ sample size estimation for other treatments not relevant for the present review, ^4^ sample size estimation for triglycerides, ^5^ sample size estimation for systolic blood pressure, ^6^ results on nitric oxide only published for baseline and 2 h values, ^7^ AUCs for appetite sensations not mentioned, ^8^ systolic and diastolic blood pressure and species derived from nitric oxide not mentioned, ^9^ results on some biomarkers not mentioned in registration, e.g., advanced glycation end products and endothelin-1; some registered biomarkers not published, e.g., asymmetric dimethyl arginine, ^10^ data on flow-mediated dilatation not available due to methodological difficulties (personal communication).

Moreover, potential confounders on the measured outcomes were only partly considered. Most studies provided instructions on nutritional behavior [20,23,24,25,28,29,30,31] and/or physical activity [20,23,24,25,27,28,29,31], however only Kirch et al. [20] and Greenberg et al. [25] checked adherence to dietary instructions by dietary records. Furthermore, only Dower et al. [28] and Kirch et al. [20] determined anthropometric parameters (BW [20,28], FM, waist circumference, WHR [20]). Compliance with interventions in long-term studies was checked by Dower et al. [28,29,31] and Kirch et al. [20].

A sample size estimation on the effect of an EC intake was performed in five studies with FMD [24,27,28], SBP [20], and TGs [30] as the primary outcome.

## 4. Discussion

### 4.1. Cardiometabolic Efficacy

To the best of our knowledge, this is the first systematic review on the effect of an EC intake on cardiometabolic parameters from RCTs. In total, the results of seven acute studies, three long-term trials, as well as from one acute-on-chronic study were considered. In contrast to our expectations, an average treatment effect was mostly not detectable.

With regard to vascular function, effects were mostly found after an acute EC intake, but only with regard to the FMD [22,27] and PAT index [22], which reflect the elasticity of macro- and microvessels, respectively. Two [22,27] out of four [22,24,26,27] acute studies observed an increase in FMD after an intake of 0.5 mg EC/kg BW [27], 1.0 mg EC/kg BW [22,27], and 2.0 mg EC/kg BW [22]. No changes occurred after ingestion of 0.1 mg EC/kg BW [27], suggesting a clear dose-dependent effect. In the study of Alañón et al. [27], the changes in FMD response correlated with the changes in EC metabolites in plasma 1 h and 2 h after EC intake. On the other hand, effects on FMD in the studies of Dower et al. [24] and Ward et al. [26], providing 100 mg [24] and 200 mg [26] EC, respectively, did not occur. Contrary to Alañón et al. [27] and Schroeter et al. [22], Ward et al. [26] and Dower et al. [24] administered a fixed dose of EC. As subjects with a BMI between 18 and 35 kg/m^2^ (>20 and ≤30 kg/m^2^ [24], ≥18 and ≤35 kg/m^2^ [26]) were recruited, the dose of 100 mg [24] and 200 mg EC [26] corresponded to an amount of 1.1 to 1.6 mg EC kg/BW [24] and 1.9 to 3.6 g EC kg/BW [26], respectively, if a body height of 1.75 m is assumed. These doses per kg BW were above those which increased FMD in the study of Alañón et al. [27] and Schroeter et al. [22]. However, both studies which observed favorable effects [22,27] differ from the other two in other respects [24,26]. First, FMD increased 1 h and/or 2 h after intervention [22,27] and no effects were found after 4 h and 6 h [27]. The maximum concentration of epicatechin metabolites in plasma occurred 1 h after ingestion of an aqueous solution of [2-^14^C] EC in a fasting state considering radioactivity, while the individual metabolites determined by HPLC-MS peaked after 1.3–2.6 h [32]. Ward et al. [26] measured FMD only 1 h and 4 h after EC intake. Furthermore, EC was ingested postprandially after a breakfast; thus, it is conceivable that the absorption and consequently the effect of EC was slowed down by the food matrix. In consequence, effects that might have occurred after 2 h were not detected. Dower et al. [24] measured parameters of vascular function 2 h after administration of encapsulated EC together with white chocolate when the sum of glucuronidated, sulfated, and methylated epicatechin metabolites in plasma also achieved maximum concentration. Second, it should be noted that the subjects of both acute studies without effects on FMD were much older (62 ± 9 y [24], 60 ± 8 y [26]; means ± SDs) than those of Schroeter et al. (25–32 y, range [22]) and Alañón et al. (23 ± 6 y, mean ± SD [27]), which detected an increase in FMD. Due to an age-related decline in FMD, which is partly ascribed to diminished smooth muscle responsiveness [33], the response on EC intake might be lower in older than in younger adults.

Long-term effects of an EC intake on FMD were only investigated by Dower et al. [28]. As plasma epicatechin metabolites were only detectable in low concentrations 8 h after EC administration [24,34] and the significance of microbial degradation products (about 95% of all epicatechin metabolites after 12 h [34]) of EC have not been investigated yet, the absence of effects on FMD in fasting state after a 4 wk intake of EC seems plausible. The same subjects received an acute dose of EC after completing the 4 wk intervention (acute-on-chronic study). Since no effect was observed in subjects with a mean age about 60 y 2 h after an acute intake of 100 mg EC [24], the lack of effects after acute-on-chronic administration of 100 mg EC is not surprising either [28].

Further parameters of vascular function remained unchanged after acute (SBP, DBP [24,26,27], AIx_75_, SEVR, ejection duration [24], LDI [27]), chronic (SBP, DBP [20,28,30], PWV, AIx_75_, EID, SEVR [28]) and acute-on-chronic (EID [28]) EC intake. As it can be implied from the proposed mechanisms of action, an increased NO availability appears to be the underlying mechanism of action of EC on vascular parameters; additionally, a decrease in the vasoconstrictive ET-1 is discussed. Consistent with the results of the vascular parameters, changes in the parameters of NO availability (plasma: NOx [24,27,28], nitrite [26,27], nitrate [23,27], and urine: nitrite [23]), as well as in ET-1 (plasma [24,28], urine [23]) after EC intake were not observed. Kirch et al. [20] found no changes in the concentration of arginine, a substrate of eNOS. This suggests that beneficial effects of EC on FMD and PAT index may not be exclusively mediated by NO and that other mechanisms of actions may have contributed to the significant increases in FMD. Considering Loke et al. [23], the intake of 200 mg EC increased 2 h plasma SNO and 5 h total urine nitrate, while ET-1 in 2 h plasma decreased. In this case, an improvement in vascular function can be assumed, but unfortunately the parameters of vascular function (e.g., FMD, BP) were not measured.

Glucose and lipid metabolism were only investigated in long-term studies [20,28,30,31]. Effects were only observed by Dower et al. [28] (reduction in insulin and HOMA-IR) which, however, disappear if data were corrected for multiple comparisons. Gutiérrez-Salmeán et al. [30], who provided also 100 mg/d EC for 4 wk as Dower et al., did not find any differences between EC and placebo treatment with regard to TGs, TC, LDL-C, TGs/HDL-C, and non-HDL-C (all *p* > 0.05). In contrast to Gutiérrez-Salmeán et al. [30], the subjects of Dower et al. [28] were metabolically healthy. Not all participants of Kirch et al. [20] were hypertriglyceridemic. Additionally, they ingested only 25 mg/d EC for only 2 wk.

The RCTs showed no changes in markers of oxidative stress [20,23], suggesting that the antioxidant potential of EC metabolites at the concentrations occurring in plasma after an acute EC ingestion [23] and after 12 h overnight fast following a chronic EC intake [20] is low or does not contribute to protection against lipid peroxidation.

For most biomarkers of inflammation, no effects were detectable either [29]. In the trial of Dower et al. [29], only sE-selectin decreased after EC administration, but significance was missing after Bonferroni correction for multiple testing. However, an additional analysis of gene expression profiles in peripheral blood mononuclear cells obtained from the study of Dower et al. [28] showed a decreased expression of gene sets involved in inflammation [35].

The influence of EC on appetite sensations (crossover study) and food intake (crossover and parallel group study) was only investigated by Greenberg et al. [25]. Contrary to the expectations, a decrease in satiety and fullness was observed after EC intake, while hunger and emptiness increased. However, *ad libitum* food intake remained unchanged [25] and a chronic EC intake neither modulates BW [20,28,29,30,31] nor FM and fat distribution [20]. Hence, the effects of EC on energy intake or energy balance are rather unlikely. In contrast to cocoa, many factors affecting energy balance (e.g., skin heat dissipation) have not been investigated for EC so far.

It should be mentioned that the sample size was estimated only in six trials [20,24,25,27,28,30]; in one of them, it was related to another study arm (cocoa) and not to EC [25]. In three trials, sample size was calculated for FMD [24,27,28] and in one case each for SBP [20] and TGs [30]. Two studies with young adults demonstrated an increase in FMD after EC treatment [22,27], whereas Dower et al. [24] (sample size estimation done) and Ward et al. [26], who investigated elderly, did not. Thus, an acute EC intake seems to increase FMD in young adults, which appears rather unlikely for older ones. For chronic effects of EC intake, only a single study is available suggesting no effects on FMD [28]. Sample size estimation on TGs was only done by Gutiérrez-Salmeán et al. [30]; an average treatment effect was lacking, which was similar in studies without sample size estimation for TGs [20,28]. Of course, the absence of significant changes in the studies of Kirch et al. [20] and Dower et al. [28] does not confirm the lack of effects. If an acute EC intake modulates SBP remains open, as SBP was only a secondary parameter in RCTs which did not find any changes [24,26,27]. For chronic EC intake, an impact on SBP is rather unlikely as Kirch et al. [20] did not find any changes; on the other hand, this remains open for Gutiérrez-Salmeán et al. [30] and Dower et al. [28]. Finally, for other parameters on cardiometabolic health that were only secondary outcome markers, it remains open if there is no effect or if this was not detectable due to an insufficient sample size.

### 4.2. (−)-Epicatechin—Not (Solely) Responsible for the Well-Known Effects of Cocoa?

Contrary to cocoa studies, the beneficial effects of an EC intake on cardiovascular parameters were only partly detectable. This is surprising, as growing evidence exists that cocoa-rich foods improve the parameters of cardiovascular health [2,3,4,5,6,7] and EC is discussed as a functional ingredient of cocoa. First, the amount of EC used in the RCTs mostly exceeded the amount of EC in a usual serving size of cocoa products. For example, 100 mg EC provided in several EC studies (Table 2 and Table 3) can be achieved by large amounts of cocoa-rich foods: 50–100 g cocoa powder and 50–200 g dark chocolate [36]. Second, the duration of EC long-term studies (2–4 wk) was similar to those of a subgroup of cocoa studies with an intervention period <3 wk, where a treatment effect was observed for FMD, SBP, DBP, FPG, TC, and LDL-C [2]. Therefore, against our previous expectations, it has to be assumed that EC is not solely responsible for the well-known effects of cocoa. A recently published RCT of Sansone et al. [37] revealed that an acute ingestion of pure EC (75 mg) together with methylxanthines (MX; 91.7% theobromine, 8.3% caffeine) increased the bioavailability of EC by 22% (AUC_0–4h_) and also the plasma concentrations of EC metabolites after 1 and 2 h compared to the administration of EC without MX. Moreover, combined intake of CF with MX had stronger effects on FMD and PWV than CF without MX, whereas MX alone did not affect both outcomes. In addition, the intake of CF with MX lowered DBP compared to the intake of CF alone. Hence, MX in cocoa may contribute to the effect of cocoa by increasing the bioavailability of EC. This might explain why bolus ingestion of pure EC did not exert similar cardiovascular effects as observed after acute cocoa consumption.

Hence, other ingredients than EC (or in addition to EC) could be relevant for the effects of cocoa. Theobromine treatment for 4 wk (500 mg/d [38], 850 mg/d [39]) has shown to improve serum lipids (increase in HDL-C and apolipoprotein (Apo) AI [39], decrease in TC [38], LDL-C, and ApoB [38,39]) vs. placebo, but not after enrichment of cocoa with 850 mg theobromine [39]. A further RCT determined the effects of an intake of cocoa extract providing CF either with DP of 1–10 (130 mg/d EC, 560 mg/d procyanidins) or with DP of 2–10 (20 mg/d EC, 540 mg/d procyanidins) on serum lipids compared to a flavanol-free control. All treatments lasted one month and were matched for theobromine and caffeine. As TC decreased after daily intake of both DP1–10 and DP2–10 as compared with the control, the reduction in TC seems to be linked to ingestion of procyanidins, but not necessarily to that of EC [40].

### 4.3. Study Quality and Consideration of Potential Confounders

Most trials were robust RCTs, which are considered as a gold standard for effectiveness research, and which imply blinding, concealment of allocation, intention-to-treat analysis, and a sufficiently large sample size [41]. All studies except of two [25,30] had a crossover design [22,23,24,25,26,27,28,29,31]. Hence, disparities in the effects due to interindividual differences can be ruled out.

Moreover, original contributions mostly fulfilled the Consolidated Standards of Reporting Trials (CONSORT) [42], but some details in allocation concealment were not reported [20,22,23,24,25,26,27,30] or a study registration was lacking [22,23,25,26,27,30]. This explains why the RoB for the outcomes of the included studies was always rated with some concerns; only the results of Dower et al. were judged with low risk (Figure 2). Furthermore, potential confounders that might bias outcomes were adequately considered in most studies, which strengthened the validity of their results (Table 4). Although adherence to instructions on dietary intake and physical activity was only checked by Greenberg et al. [25] and Kirch et al. [20], at least for dietary intake, confounding effects by lifestyle is rather unlikely. The results of the studies appear to be valid whether or not effects were observed. However, as most outcomes were only secondary parameters, a final conclusion of whether there are no effects cannot be drawn.

### 4.4. Strengths and Limitations

A strength of this systematic review is the application of the revised Cochrane RoB 2 tool and the consideration of additional factors which might have biased the investigated outcomes. Moreover, two databases, PubMed and Web of Science, were used for literature search. Whether an extension of the literature search to paid databases such as EMBASE and Scopus would reveal further studies of relevance seems rather unlikely, especially since the reference lists of the included studies were checked for potentially relevant RCTs.

The inclusion of studies with heterogeneous target groups may be considered as a weakness, but a stricter selection was not possible due to the limited number of studies on this topic of research.

## 5. Conclusions

An acute EC intake may improve endothelial function of micro- and/or macrovessels in healthy young adults in a dose-dependent manner (0.5–2.0 mg/kg BW). As most cardiometabolic parameters that remained unchanged by EC treatment were only secondary outcomes (e.g., inflammation, oxidative stress), a final conclusion on the lack of effects cannot be drawn so far. Thus, robust studies on the effect of an EC intake which ensure a low RoB are needed. As it seems to be conceivable that cocoa ingredients beyond EC might contribute to the cardioprotective effects of cocoa, and additive or synergistic effects should be investigated in future studies.

## Data Availability

Not applicable.
